# (2*E*)-3-[4-(Benz­yloxy)phen­yl]-1-(pyridin-3-yl)prop-2-en-1-one

**DOI:** 10.1107/S1600536812034897

**Published:** 2012-08-15

**Authors:** Hoong-Kun Fun, Ching Kheng Quah, Prakash S. Nayak, B. Narayana, B. K. Sarojini

**Affiliations:** a X-ray Crystallography Unit, School of Physics, Universiti Sains Malaysia, 11800 USM, Penang, Malaysia; b Department of Studies in Chemistry, Mangalore University, Mangalagangotri 574 199, India; c Department of Chemistry, P. A. College of Engineering, Nadupadavu, Mangalore 574 153, India

## Abstract

The title compound, C_21_H_17_NO_2_, exists in an *E* conformation with respect to the C=C bond. The pyridine ring forms dihedral angles of 5.57 (7) and 82.30 (9)°, respectively, with the central benzene ring and the terminal phenyl ring. The dihedral angle between the benzene and phenyl rings is 87.69 (8)°. No significant inter­molecular inter­actions are observed.

## Related literature

For the pharmacological activity of chalcones, see: Matsuda *et al.* (2003 ▶); Lopez *et al.* (2001 ▶); Agarwal *et al.* (2005 ▶). For related structures, see: Bibila Mayaya Bisseyou *et al.* (2007 ▶); Liu *et al.* (2005 ▶); Jasinski *et al.* (2011 ▶). For the stability of the temperature controller used in the data collection, see: Cosier & Glazer (1986 ▶).
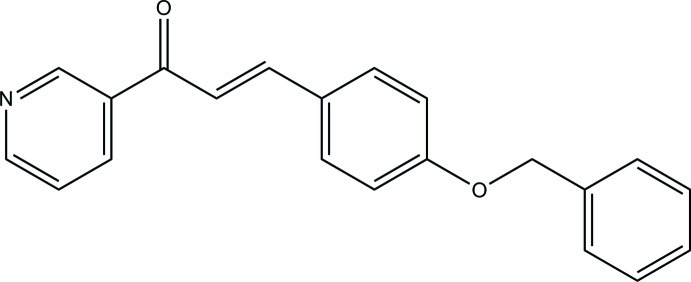


## Experimental

### 

#### Crystal data

C_21_H_17_NO_2_
*M* *_r_* = 315.36
Monoclinic, 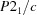

*a* = 5.9845 (6) Å
*b* = 38.187 (4) Å
*c* = 8.5412 (7) Å
β = 123.372 (5)°
*V* = 1630.1 (3) Å^3^
*Z* = 4
Mo *K*α radiation
μ = 0.08 mm^−1^
*T* = 100 K
0.40 × 0.33 × 0.17 mm

#### Data collection

Bruker SMART APEXII DUO CCD area-detector diffractometer
Absorption correction: multi-scan (*SADABS*; Bruker, 2009 ▶) *T*_min_ = 0.968, *T*_max_ = 0.986
18812 measured reflections
4751 independent reflections
3171 reflections with *I* > 2σ(*I*)
*R*_int_ = 0.028

#### Refinement

*R*[*F* ^2^ > 2σ(*F* ^2^)] = 0.050
*wR*(*F* ^2^) = 0.141
*S* = 1.02
4751 reflections
217 parameters
H-atom parameters constrained
Δρ_max_ = 0.17 e Å^−3^
Δρ_min_ = −0.15 e Å^−3^

### 

Data collection: *APEX2* (Bruker, 2009 ▶); cell refinement: *SAINT* (Bruker, 2009 ▶); data reduction: *SAINT*; program(s) used to solve structure: *SHELXTL* (Sheldrick, 2008 ▶); program(s) used to refine structure: *SHELXTL*; molecular graphics: *SHELXTL*; software used to prepare material for publication: *SHELXTL* and *PLATON* (Spek, 2009 ▶).

## Supplementary Material

Crystal structure: contains datablock(s) global, I. DOI: 10.1107/S1600536812034897/is5181sup1.cif

Structure factors: contains datablock(s) I. DOI: 10.1107/S1600536812034897/is5181Isup2.hkl

Supplementary material file. DOI: 10.1107/S1600536812034897/is5181Isup3.cml

Additional supplementary materials:  crystallographic information; 3D view; checkCIF report

## Figures and Tables

**Fig. 1.**
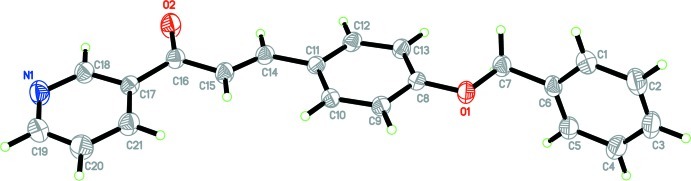
The molecular structure of the title compound showing 30% probability displacement ellipsoids for non-H atoms.

## supplementary crystallographic information

### Comment

Chalcones constitute an important family of substances belonging to flavonoids and isoflavonoids and are abundant in edible plants. Chalcones exhibit many pharmacological activities, including anti-leishmanial, anti-inflammatory (Matsuda *et al.*, 2003), anti-mitotic, anti-invasive, anti-tuberculosis, anti-fungal (Lopez *et al.*, 2001) and anti-malarial (Agarwal *et al.*, 2005). Nitrogen moiety containing heterocyclic chalcones plays important roles as anti-ulcer, herbicidal, anti-bacterial, analgesic, sedative, anti-phlogistic and virucidal agents. The crystal structures of some chalcones derived from acetyl pyridine viz., (*Z*)-3-(2,6-dichlorophenyl)-1-(pyridin-3-yl)-2- (1H-1,2,4-triazol-1-yl)prop-2-en-1-one (Liu *et al.*, 2005), 3-(3-chlorophenyl)-1-(2-methylimidazo[1,2-a]pyridin-3-yl)prop-2-en-1-one (Bibila Mayaya Bisseyou *et al.*, 2007) and (2*E*)-3-(3-bromo-4-methoxyphenyl)-1-(pyridin-2-yl)prop-2-en-1-one (Jasinski *et al.*, 2011) have been reported. In continuation of our studies on chalcones and their derivatives, the title compound (I) was prepared and its crystal structure is reported.

The title compound (Fig. 1) exists in an *E* configuration with respect to the C14═C15 bond [1.3217 (19) Å]. The pyridin-3-yl ring (N1/C17–C21) forms dihedral angles of 5.57 (7) and 82.30 (9)° with the benzene (C8–C13) and phenyl (C1–C6) rings, respectively. The dihedral angle between the benzene and phenyl rings is 87.69 (8)°. Bond lengths and angles are within normal ranges and are comparable to related structures (Bibila Mayaya Bisseyou *et al.*, 2007; Liu *et al.*, 2005; Jasinski *et al.*, 2011). No significant intermolecular hydrogen bonds are observed.

### Experimental

To a mixture of 3-acetylpyridine (1.1 mL, 0.01 mol) and 4-benzyloxybenzaldehyde (2.12 g, 0.01 mol) in ethanol (100 mL), 15 mL of 10% sodium hydroxide solution was added and stirred at 0–5 °C for 3 h. The precipitate formed was collected by filtration and purified by recrystallization from ethanol. Single crystals were grown from acetone and toluene (1:1) mixture by slow evaporation method (*m.p.* 403–407 K).

### Refinement

All H atoms were positioned geometrically and refined using a riding model with C—H = 0.95 or 0.99 Å and *U*_iso_(H) = 1.2 or 1.5*U*_eq_(C).

### Crystal data

**Table:**

C_21_H_17_NO_2_	*F*(000) = 664
*M**_r_* = 315.36	*D*_x_ = 1.285 Mg m^−^^3^
Monoclinic, *P*2_1_/*c*	Mo *K*α radiation, λ = 0.71073 Å
Hall symbol: -P 2ybc	Cell parameters from 4372 reflections
*a* = 5.9845 (6) Å	θ = 2.9–26.5°
*b* = 38.187 (4) Å	µ = 0.08 mm^−^^1^
*c* = 8.5412 (7) Å	*T* = 100 K
β = 123.372 (5)°	Block, yellow
*V* = 1630.1 (3) Å^3^	0.40 × 0.33 × 0.17 mm
*Z* = 4	

### Data collection

**Table:**

Bruker SMART APEXII DUO CCD area-detector diffractometer	4751 independent reflections
Radiation source: fine-focus sealed tube	3171 reflections with *I* > 2σ(*I*)
Graphite monochromator	*R*_int_ = 0.028
φ and ω scans	θ_max_ = 30.0°, θ_min_ = 2.1°
Absorption correction: multi-scan (*SADABS*; Bruker, 2009)	*h* = −8→8
*T*_min_ = 0.968, *T*_max_ = 0.986	*k* = −53→47
18812 measured reflections	*l* = −11→11

### Refinement

**Table:**

Refinement on *F*^2^	Primary atom site location: structure-invariant direct methods
Least-squares matrix: full	Secondary atom site location: difference Fourier map
*R*[*F*^2^ > 2σ(*F*^2^)] = 0.050	Hydrogen site location: inferred from neighbouring sites
*wR*(*F*^2^) = 0.141	H-atom parameters constrained
*S* = 1.02	*w* = 1/[σ^2^(*F*_o_^2^) + (0.059*P*)^2^ + 0.2532*P*] where *P* = (*F*_o_^2^ + 2*F*_c_^2^)/3
4751 reflections	(Δ/σ)_max_ = 0.001
217 parameters	Δρ_max_ = 0.17 e Å^−^^3^
0 restraints	Δρ_min_ = −0.15 e Å^−^^3^

### Special details

**Table:**

Experimental. The crystal was placed in the cold stream of an Oxford Cryosystems Cobra open-flow nitrogen cryostat (Cosier & Glazer, 1986) operating at 100.0 (1) K.
Geometry. All esds (except the esd in the dihedral angle between two l.s. planes) are estimated using the full covariance matrix. The cell esds are taken into account individually in the estimation of esds in distances, angles and torsion angles; correlations between esds in cell parameters are only used when they are defined by crystal symmetry. An approximate (isotropic) treatment of cell esds is used for estimating esds involving l.s. planes.
Refinement. Refinement of F^2^ against ALL reflections. The weighted R-factor wR and goodness of fit S are based on F^2^, conventional R-factors R are based on F, with F set to zero for negative F^2^. The threshold expression of F^2^ > 2sigma(F^2^) is used only for calculating R-factors(gt) etc. and is not relevant to the choice of reflections for refinement. R-factors based on F^2^ are statistically about twice as large as those based on F, and R- factors based on ALL data will be even larger.

### Fractional atomic coordinates and isotropic or equivalent isotropic displacement parameters (Å^2^)

**Table:**

	*x*	*y*	*z*	*U*_iso_*/*U*_eq_	
O1	−0.1642 (2)	−0.12817 (2)	0.76557 (15)	0.0534 (3)	
O2	0.6625 (3)	0.04322 (3)	0.69065 (19)	0.0690 (3)	
N1	0.5921 (3)	0.14650 (3)	0.7813 (2)	0.0668 (4)	
C1	−0.2000 (4)	−0.20724 (4)	0.8950 (3)	0.0637 (4)	
H1A	−0.0178	−0.2048	0.9949	0.076*	
C2	−0.3631 (4)	−0.23073 (4)	0.9115 (3)	0.0725 (5)	
H2A	−0.2924	−0.2442	1.0225	0.087*	
C3	−0.6271 (4)	−0.23442 (4)	0.7671 (3)	0.0700 (5)	
H3A	−0.7382	−0.2506	0.7778	0.084*	
C4	−0.7299 (4)	−0.21464 (4)	0.6074 (3)	0.0673 (4)	
H4A	−0.9128	−0.2169	0.5085	0.081*	
C5	−0.5668 (3)	−0.19132 (4)	0.5905 (2)	0.0583 (4)	
H5A	−0.6381	−0.1779	0.4792	0.070*	
C6	−0.3010 (3)	−0.18749 (3)	0.7344 (2)	0.0496 (3)	
C7	−0.1254 (3)	−0.16226 (3)	0.7147 (2)	0.0524 (3)	
H7A	0.0645	−0.1693	0.7978	0.063*	
H7B	−0.1734	−0.1621	0.5838	0.063*	
C8	−0.0323 (3)	−0.10096 (3)	0.74586 (18)	0.0425 (3)	
C9	−0.0638 (3)	−0.06836 (3)	0.8049 (2)	0.0480 (3)	
H9A	−0.1686	−0.0664	0.8569	0.058*	
C10	0.0551 (3)	−0.03911 (3)	0.7886 (2)	0.0467 (3)	
H10A	0.0300	−0.0170	0.8282	0.056*	
C11	0.2133 (2)	−0.04140 (3)	0.71422 (18)	0.0427 (3)	
C12	0.2424 (3)	−0.07406 (3)	0.65656 (19)	0.0485 (3)	
H12A	0.3472	−0.0761	0.6045	0.058*	
C13	0.1238 (3)	−0.10387 (4)	0.6723 (2)	0.0484 (3)	
H13A	0.1490	−0.1260	0.6332	0.058*	
C14	0.3499 (3)	−0.01114 (3)	0.69900 (19)	0.0467 (3)	
H14A	0.4507	−0.0153	0.6454	0.056*	
C15	0.3490 (3)	0.02140 (4)	0.7514 (2)	0.0507 (3)	
H15A	0.2462	0.0269	0.8021	0.061*	
C16	0.5021 (3)	0.04934 (4)	0.73355 (19)	0.0470 (3)	
C17	0.4655 (3)	0.08627 (3)	0.77386 (18)	0.0433 (3)	
C18	0.6038 (3)	0.11266 (4)	0.7499 (2)	0.0562 (4)	
H18A	0.7154	0.1060	0.7079	0.067*	
C19	0.4329 (4)	0.15510 (4)	0.8397 (2)	0.0652 (4)	
H19A	0.4199	0.1791	0.8629	0.078*	
C20	0.2878 (4)	0.13133 (4)	0.8679 (3)	0.0687 (5)	
H20A	0.1783	0.1388	0.9103	0.082*	
C21	0.3026 (3)	0.09635 (4)	0.8339 (2)	0.0579 (4)	
H21A	0.2022	0.0794	0.8516	0.069*	

### Atomic displacement parameters (Å^2^)

**Table:**

	*U*^11^	*U*^22^	*U*^33^	*U*^12^	*U*^13^	*U*^23^
O1	0.0640 (6)	0.0392 (5)	0.0748 (7)	−0.0061 (4)	0.0494 (6)	−0.0041 (4)
O2	0.0864 (8)	0.0557 (6)	0.1028 (9)	−0.0082 (5)	0.0761 (8)	−0.0071 (6)
N1	0.0781 (10)	0.0477 (7)	0.0865 (10)	−0.0058 (6)	0.0528 (9)	0.0013 (6)
C1	0.0684 (10)	0.0500 (8)	0.0743 (10)	0.0020 (7)	0.0402 (9)	0.0058 (7)
C2	0.0935 (14)	0.0524 (9)	0.0850 (12)	0.0029 (9)	0.0576 (12)	0.0134 (8)
C3	0.0904 (13)	0.0484 (8)	0.0985 (13)	−0.0127 (8)	0.0693 (12)	−0.0046 (8)
C4	0.0667 (10)	0.0617 (9)	0.0824 (12)	−0.0148 (8)	0.0466 (9)	−0.0097 (8)
C5	0.0647 (10)	0.0507 (8)	0.0662 (9)	−0.0047 (7)	0.0403 (8)	−0.0012 (7)
C6	0.0615 (9)	0.0347 (6)	0.0651 (9)	−0.0013 (6)	0.0427 (8)	−0.0047 (6)
C7	0.0595 (9)	0.0414 (7)	0.0660 (9)	−0.0045 (6)	0.0407 (8)	−0.0062 (6)
C8	0.0407 (7)	0.0410 (6)	0.0458 (7)	−0.0022 (5)	0.0237 (6)	0.0015 (5)
C9	0.0504 (8)	0.0456 (7)	0.0593 (8)	0.0005 (6)	0.0372 (7)	0.0008 (6)
C10	0.0479 (7)	0.0395 (6)	0.0576 (8)	0.0009 (5)	0.0321 (7)	0.0009 (5)
C11	0.0384 (6)	0.0453 (7)	0.0424 (6)	−0.0012 (5)	0.0209 (6)	0.0035 (5)
C12	0.0489 (8)	0.0525 (7)	0.0535 (8)	−0.0048 (6)	0.0342 (7)	−0.0043 (6)
C13	0.0510 (8)	0.0446 (7)	0.0555 (8)	−0.0037 (6)	0.0330 (7)	−0.0067 (6)
C14	0.0465 (7)	0.0492 (7)	0.0482 (7)	−0.0024 (6)	0.0284 (6)	0.0036 (6)
C15	0.0560 (8)	0.0477 (7)	0.0596 (8)	−0.0048 (6)	0.0390 (7)	0.0015 (6)
C16	0.0510 (8)	0.0485 (7)	0.0486 (7)	−0.0030 (6)	0.0320 (6)	0.0021 (6)
C17	0.0433 (7)	0.0467 (7)	0.0409 (6)	−0.0022 (5)	0.0238 (6)	0.0033 (5)
C18	0.0607 (9)	0.0508 (8)	0.0707 (10)	−0.0041 (6)	0.0447 (8)	0.0024 (7)
C19	0.0721 (11)	0.0509 (8)	0.0724 (11)	−0.0003 (7)	0.0397 (9)	−0.0062 (7)
C20	0.0771 (11)	0.0644 (10)	0.0856 (12)	−0.0041 (8)	0.0582 (10)	−0.0135 (8)
C21	0.0640 (9)	0.0580 (8)	0.0682 (9)	−0.0079 (7)	0.0469 (8)	−0.0041 (7)

### Geometric parameters (Å, º)

**Table:**

O1—C8	1.3700 (15)	C9—H9A	0.9500
O1—C7	1.4313 (15)	C10—C11	1.4037 (18)
O2—C16	1.2235 (16)	C10—H10A	0.9500
N1—C18	1.3297 (19)	C11—C12	1.3869 (18)
N1—C19	1.337 (2)	C11—C14	1.4620 (17)
C1—C6	1.380 (2)	C12—C13	1.3866 (18)
C1—C2	1.388 (2)	C12—H12A	0.9500
C1—H1A	0.9500	C13—H13A	0.9500
C2—C3	1.376 (3)	C14—C15	1.3217 (19)
C2—H2A	0.9500	C14—H14A	0.9500
C3—C4	1.373 (3)	C15—C16	1.4682 (18)
C3—H3A	0.9500	C15—H15A	0.9500
C4—C5	1.386 (2)	C16—C17	1.4963 (18)
C4—H4A	0.9500	C17—C21	1.3826 (19)
C5—C6	1.383 (2)	C17—C18	1.3889 (18)
C5—H5A	0.9500	C18—H18A	0.9500
C6—C7	1.5019 (18)	C19—C20	1.366 (2)
C7—H7A	0.9900	C19—H19A	0.9500
C7—H7B	0.9900	C20—C21	1.380 (2)
C8—C13	1.3877 (18)	C20—H20A	0.9500
C8—C9	1.3943 (18)	C21—H21A	0.9500
C9—C10	1.3718 (18)		
			
C8—O1—C7	116.85 (10)	C11—C10—H10A	119.6
C18—N1—C19	116.22 (13)	C12—C11—C10	117.68 (11)
C6—C1—C2	120.41 (17)	C12—C11—C14	119.58 (12)
C6—C1—H1A	119.8	C10—C11—C14	122.73 (12)
C2—C1—H1A	119.8	C13—C12—C11	122.20 (12)
C3—C2—C1	119.97 (16)	C13—C12—H12A	118.9
C3—C2—H2A	120.0	C11—C12—H12A	118.9
C1—C2—H2A	120.0	C12—C13—C8	119.10 (12)
C4—C3—C2	120.02 (15)	C12—C13—H13A	120.4
C4—C3—H3A	120.0	C8—C13—H13A	120.4
C2—C3—H3A	120.0	C15—C14—C11	127.31 (12)
C3—C4—C5	120.03 (17)	C15—C14—H14A	116.3
C3—C4—H4A	120.0	C11—C14—H14A	116.3
C5—C4—H4A	120.0	C14—C15—C16	121.93 (13)
C6—C5—C4	120.44 (16)	C14—C15—H15A	119.0
C6—C5—H5A	119.8	C16—C15—H15A	119.0
C4—C5—H5A	119.8	O2—C16—C15	121.97 (13)
C1—C6—C5	119.13 (14)	O2—C16—C17	119.18 (12)
C1—C6—C7	120.75 (14)	C15—C16—C17	118.83 (11)
C5—C6—C7	120.12 (13)	C21—C17—C18	116.88 (13)
O1—C7—C6	108.00 (11)	C21—C17—C16	124.77 (12)
O1—C7—H7A	110.1	C18—C17—C16	118.36 (11)
C6—C7—H7A	110.1	N1—C18—C17	124.98 (14)
O1—C7—H7B	110.1	N1—C18—H18A	117.5
C6—C7—H7B	110.1	C17—C18—H18A	117.5
H7A—C7—H7B	108.4	N1—C19—C20	123.70 (15)
O1—C8—C13	124.89 (11)	N1—C19—H19A	118.2
O1—C8—C9	115.53 (11)	C20—C19—H19A	118.2
C13—C8—C9	119.58 (12)	C19—C20—C21	119.08 (15)
C10—C9—C8	120.64 (12)	C19—C20—H20A	120.5
C10—C9—H9A	119.7	C21—C20—H20A	120.5
C8—C9—H9A	119.7	C20—C21—C17	119.14 (14)
C9—C10—C11	120.79 (12)	C20—C21—H21A	120.4
C9—C10—H10A	119.6	C17—C21—H21A	120.4
			
C6—C1—C2—C3	0.0 (3)	C11—C12—C13—C8	0.9 (2)
C1—C2—C3—C4	−0.6 (3)	O1—C8—C13—C12	178.71 (12)
C2—C3—C4—C5	0.9 (3)	C9—C8—C13—C12	−0.9 (2)
C3—C4—C5—C6	−0.7 (2)	C12—C11—C14—C15	−178.19 (14)
C2—C1—C6—C5	0.2 (2)	C10—C11—C14—C15	0.7 (2)
C2—C1—C6—C7	−179.44 (14)	C11—C14—C15—C16	178.09 (13)
C4—C5—C6—C1	0.1 (2)	C14—C15—C16—O2	−8.9 (2)
C4—C5—C6—C7	179.77 (13)	C14—C15—C16—C17	172.68 (13)
C8—O1—C7—C6	−176.20 (12)	O2—C16—C17—C21	−176.13 (15)
C1—C6—C7—O1	−96.55 (15)	C15—C16—C17—C21	2.4 (2)
C5—C6—C7—O1	83.83 (16)	O2—C16—C17—C18	3.9 (2)
C7—O1—C8—C13	3.3 (2)	C15—C16—C17—C18	−177.67 (13)
C7—O1—C8—C9	−177.05 (12)	C19—N1—C18—C17	−0.2 (3)
O1—C8—C9—C10	−178.79 (12)	C21—C17—C18—N1	0.4 (2)
C13—C8—C9—C10	0.9 (2)	C16—C17—C18—N1	−179.58 (15)
C8—C9—C10—C11	−0.7 (2)	C18—N1—C19—C20	0.2 (3)
C9—C10—C11—C12	0.6 (2)	N1—C19—C20—C21	−0.4 (3)
C9—C10—C11—C14	−178.24 (13)	C19—C20—C21—C17	0.5 (3)
C10—C11—C12—C13	−0.7 (2)	C18—C17—C21—C20	−0.5 (2)
C14—C11—C12—C13	178.21 (13)	C16—C17—C21—C20	179.44 (15)
